# Ultraviolet luminosity density of the universe during the epoch of reionization

**DOI:** 10.1038/ncomms8945

**Published:** 2015-09-08

**Authors:** Ketron Mitchell-Wynne, Asantha Cooray, Yan Gong, Matthew Ashby, Timothy Dolch, Henry Ferguson, Steven Finkelstein, Norman Grogin, Dale Kocevski, Anton Koekemoer, Joel Primack, Joseph Smidt

**Affiliations:** 1 Department of Physics & Astronomy, University of California, Irvine, California 92697, USA; 2 National Astronomical Observatories, Chinese Academy of Sciences, 20A Datun Road, Chaoyang District, Beijing 100012, China; 3 Harvard-Smithsonian Center for Astrophysics, 60 Garden St., Cambridge, Massachusetts 02138, USA; 4 Department of Astronomy, Cornell University, Ithaca, New York 14853, USA; 5 Space Telescope Science Institute, 3700 San Martin Dr., Baltimore, Maryland 21218, USA; 6 Department of Astronomy, The University of Texas at Austin, Austin, Texas 78712, USA; 7 Department of Physics and Astronomy, University of Kentucky, Lexington, Kentucky 40506, USA; 8 Physics Department, University of California Santa Cruz, Santa Cruz, California 95064, USA; 9 Theoretical Division, Los Alamos National Laboratory, Los Alamos, New Mexico 87545, USA

## Abstract

The spatial fluctuations of the extragalactic background light trace the total emission from all stars and galaxies in the Universe. A multiwavelength study can be used to measure the integrated emission from first galaxies during reionization when the Universe was about 500 million years old. Here we report arcmin-scale spatial fluctuations in one of the deepest sky surveys with the Hubble Space Telescope in five wavebands between 0.6 and 1.6 μm. We model-fit the angular power spectra of intensity fluctuation measurements to find the ultraviolet luminosity density of galaxies at redshifts greater than 8 to be 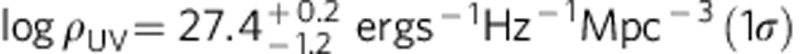. This level of integrated light emission allows for a significant surface density of fainter primeval galaxies that are below the point-source detection level in current surveys.

## Abstract (web summary)

The luminosity of ultraviolet light emitted by the first galaxies in the universe traces the formation and evolution of stars and galaxies which led to the epoch of reionization. Here the authors use data from the Hubble Space Telescope and through a model provide a bound for the total luminosity.

## Graphical abstract

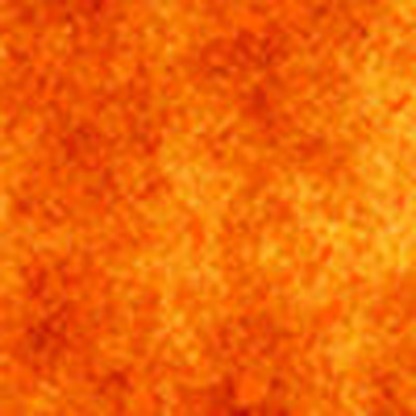

The formation and early evolution of the first galaxies in the universe occurred some time after the dark ages, when the coalescence of gravitationally bound masses formed in complex structures, with a spatial distribution that can be traced back to primordial overdensities^1^,^2^. The ultraviolet (UV) photons from these first sources initiated the reionization of the surrounding neutral medium, thus ending the dark ages and beginning the era of a transparent cosmos, which we are increasingly familiar with today. The luminosity per unit volume of these ultraviolet photons at a rest wavelength around 1,500 Å (*ρ*_UV_) during this reionization period is an important quantity to measure, as it traces the star formation and evolution of these ionizing sources. The traditional method to measure the ultraviolet luminosity density of the universe, *ρ*_UV_, during the epoch of reionization, involves searching for candidate galaxies at *z***>**6 through their Lyman-dropout signature^3^,^4^,^5^,^6^,^7^ and then constructing the luminosity function of those detected galaxies based on the observed number counts. This luminosity function is then extraploated to a fainter absolute magnitude and integrated in luminosity to calculate *ρ*_UV_.

There is a second way to quantify *ρ*_UV_. This involves a measurement of the extragalactic background light (EBL) and, in particular, the angular power spectrum of the EBL intensity fluctuations. Because these intensity fluctuations are the result of emissions throughout the cosmic time, the signal we measure today is the sum of many different emission components, from nearby in our Galaxy to distant sources. If the integrated intensity from reionization can be reliably separated from that of foreground signals, we may be able to make an accounting of the total luminosity density of UV photons from reionization. Just as Lyman-dropout galaxies are detected in deep sky surveys, there is a way to achieve such a separation. Due to redshifting of the photons arising from sources present during reionization, their emission, as seen today, is expected to peak between 0.9 and 1.1 μm. This assumes that the reionization occurred around *z*∼7 to 9, consistent with optical depth to electron scattering as measured by Planck^8^. Due to absorption of ionizing ultraviolet photons, there is no contribution shortward of the redshifted Lyman break around 0.8 μm (refs 9, 10). Spatial fluctuations of the EBL centred around 1 μm thus provide the best mechanism to discriminate the signal generated by galaxies present during reionization^11^,^12^ from those at lower redshifts, based on the strength of the drop-out signature in the fluctuations measured in different bands.

There are existing measurements of the EBL fluctuations though their origin remain uncertain. This is mostly due to the fact that the previous measurements of EBL fluctuations have until now been limited to wavelengths >1.1 μm, with the best measurements performed at 3.6 μm (refs 13, 14, 15, 16, 17). These studies have been interpreted with models involving populations of sources present during reionization at redshift *z***>**8, direct collapse and other primordial blackholes at *z***>**12 (refs 18, 19), and with stellar emission from tidally stripped intergalactic stars residing in dark matter halos, or the ‘intrahalo light' (IHL)^15^ at *z*<3. The IHL is diffuse stars in dark matter halos due to galaxy mergers and tidal interactions. While the relative strengths of these various contributions are still unknown, we expect the signal from high-redshift galaxies to be separable from low-redshift contributions, including those from faint nearby dwarf galaxies^20^, through a multiwavelength fluctuation study spanning the 1-μm range, including in the optical (
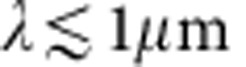
) and near-IR (
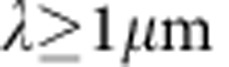
) wavelengths.

Here we present results from a multiwavelength fluctuation study using data from the Hubble Space Telescope (HST) that span across the interesting wavelength range centred at 1 μm. Through models for multiple sources of intensity fluctuations, from diffuse Galactic Light to primordial faint galaxies, we are able to describe the five-band fluctuation measurements in optical and near-infrared wavelengths to obtain a constraint on the ultraviolet luminosity density of galaxies present during reionization at a redshift above 8. We compare our measurement with existing constraints on the quantity and we also discuss the implication of our measurement.

## Results

### Fluctuation power spectra

**Figure 1:**
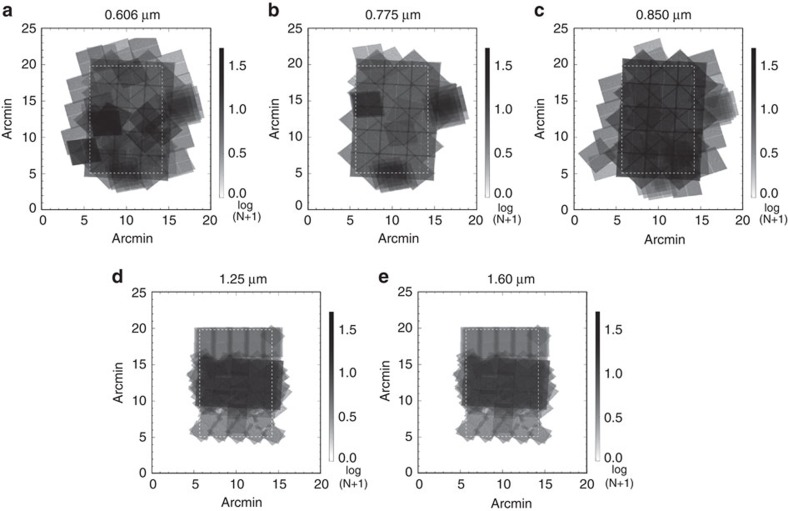
Summary of tile patterns and their data archive identifications. (**a**) Proposal ID's for each filter in the GOODS-S field. The ACS and WFC3 rows show the proposals which are common between all the bands in each instrument. For each proposal we did not necessarily use all the frames, specifically those from deep surveys. Also show are the tiling patterns for all the bands: 0.606 μm (F606W); (**b**) 0.775 μm (F775W); (**c**) 0.850 μm (F850LP); (**d**) 1.25 μm (F125W); (**e**) and 1.60 μm (F160W); (**f**) The units of the tile pattern figures are log_10_(N+1), where N is the number of frames overlapping. The dashed white line indicates the cropped region where the fluctuation analysis was performed.

**Figure 2:**
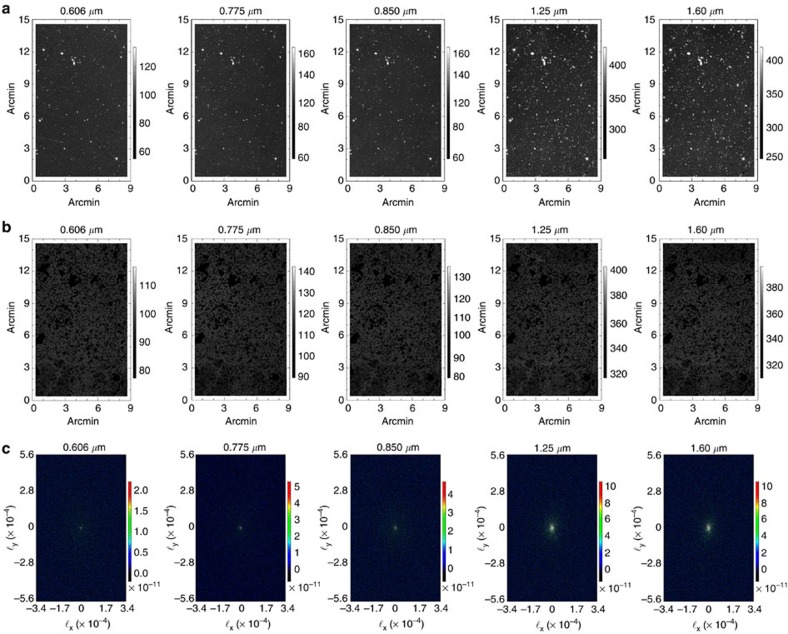
Self-calibrated mosaics. (**a**) GOODS-S SelfCal mosaics for each band. We astrometrically align each map and crop the outer regions so as to include only sections that have been observed in all five bands. The units of the maps are nW m^−2^ sr^−1^. (**b**) The same as **a** except with the source mask applied. (**c**) The fast fourier transform (FFT) of each of the maps in **b** which is what is used to measure the angular power spectrum. This is plotted in Fourier space as a function of modes ℓ_*x*_ and ℓ_*y*_. The FFT of each map is structureless and contains only Gaussian noise, which is indicative of high-quality mosaics. By definition, the units of the FFT are the same as the units of the map. Each column is filter specific, plotted as 0.606, 0.775, 0.850, 1.25 and 1.60 μm.

**Figure 3:**
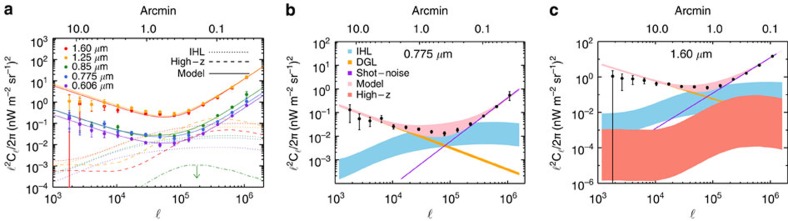
Angular power spectra of optical and near-infrared background intensity fluctuations. (**a**) Multiwavelength auto power spectra of optical to near-infrared intensity fluctuations in the GOODS-South field using Hubble Space Telescope data (see Supplementary Methods for data selection details). The error bars are calculated by adding in quadrature the errors from the beam transfer function, map-making transfer function and calibration errors, to the s.d. at each multipole, *δC*_ℓ_, described in equation 11. Thus the 1*σ* uncertainties account for all sources of noise and error, including map-making, calibration, detector noise and cosmic variance associated with finite size of the survey. We show the best-fit model which makes use of four components: (**a**) *z***>**8 high-redshift galaxies; (**b**) intrahalo light (IHL)^15^; (**c**) faint low-redshift galaxies^20^; and (**d**) diffuse Galactic light. At 1.25 and 1.6 μm, the best-fit high-redshift galaxy signal is shown as dashed lines. The signal is zero in the optical bands. We show the upper limit (denoted by a downard facing arrow) of fluctuations generated by 6<*z*<8 galaxies as a dot-dashed line. Fluctuation power spectra and the best-fit models with 1*σ* error bounds for the model components are shown at 0.775 μm in **b** and 1.60 μm in **c** The dominant model contributors to the total power spectrum are DGL at low multipoles, or angular scales greater than a few arcmin, IHL at intermediate multipoles corresponding to angular scales of about an arcmin, and shot noise associated with faint low-redshift dwarf galaxies dominating the high multipoles or sub-arcmin angular scales. The clustering signal of low-*z* galaxies is more than an order of magnitude below the lower limit plotted here, thus we did not include a low-*z* component in our modelling.

**Figure 4:**
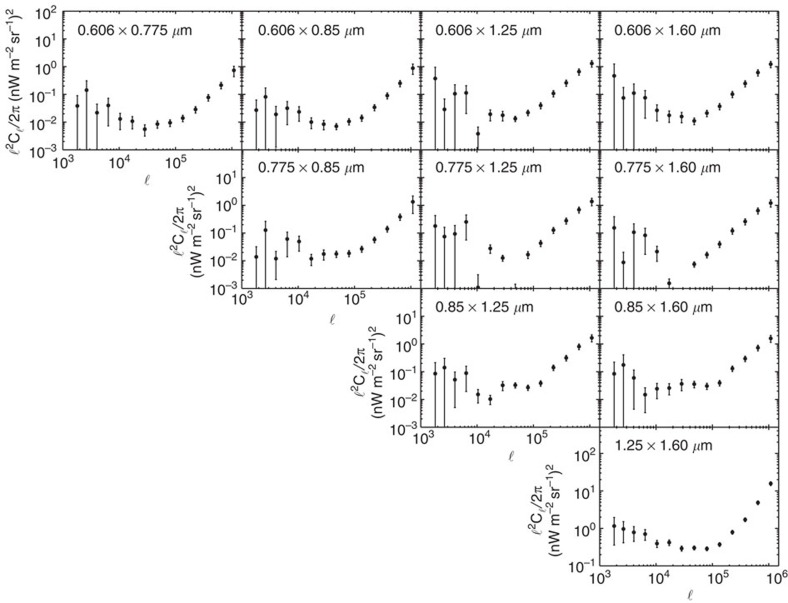
Angular cross- power spectra of optical and near-infrared background. Ten cross-correlations between the HST bands. Excess signal is detected in the cross-correlations. The error bars are 1*σ* uncertainties which are calculated in a similar way as in Fig. 3, which accounts for all sources of noise and error, including map-making, calibration, detector noise, and cosmic variance. However, the noise power spectra for the cross-correlations are calculated slightly differently. For each filter we have two maps, so for each cross-correlation between bands we have four maps (label them A and B for the first filter, and C and D for the second). This enables us to generate a noise power spectrum by computing (A–B) × (C–D), as opposed to taking the auto-spectrum of (A–B) for the autocorrelations. The first row corresponds to all correlations with the 0.606-μm band, the second for all correlations with the 0.775-μm band not found in the first row, the third row corresponds to all correlations with the 0.850-μm band not found in any of the preceding rows, and the last row corresponds to correlations at 1.25 μm. The columns similarly increase in wavelength as you move across the page.

We make use of imaging data from the Cosmic Assembly Near-Infrared Deep Extragalactic Legacy Survey^21^,^22^ (CANDELS), a legacy program of the HST (see Fig. 1). Due to extensive data in the Hubble archive, we selected the southern area of the Great Observatories Origins Deep Survey (GOODS)^23^,^24^ for the measurements (see the Methods section for details on our field selection process). This field contains HST observations with two instruments (Wide Field Camera 3 and Advanced Camera for Surveys) that have the deepest and most continuous coverage. Our total data set is comprised of observations that were taken between 2002 and 2012, with exposure times ranging between 180 and 1,469 s per frame. We avoided the background gradients evident in the publicly available WFC3 and ACS mosaics by creating our own self-calibrated mosaics using custom software^25^, to produce 120-square arcmin mosaics combined from 234 to 428 (depending on the passband) individual flux-calibrated, flat-fielded (FLT) frames (Fig. 2). These mosaics are publicly available (see Supplementary Note 1). We mask stars and galaxies using an internally developed masking algorithm, facilitated by a public multiwavelength catalogue spanning from the ultraviolet to the mid-infrared^26^. The auto- (Fig. 3) and cross-power spectra (Fig. 4) are computed using standard Fourier Transform techniques on the masked images, which retain 53% of their pixels after masking. A number of corrections are performed on the power spectra. Details regarding these corrections can be found in the Methods section.

**Figure 5:**
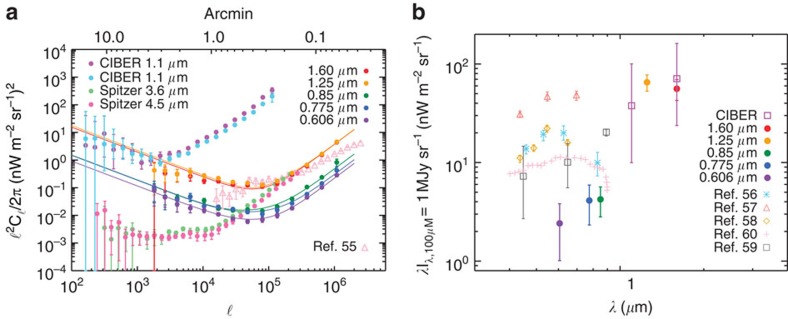
Various autospectra and the spectral energy distribution of diffuse galactic light. (**a**) CANDELS corrected power spectra plotted against the CIBER^16^, Spitzer^15^ and NICMOS^54^ measurements (see also ref. 34 for more recent NICMOS measurements). The power spectrum resulting from the NICMOS analysis was measured from a MultiDrizzle map and has not been corrected for the transfer function and mode-coupling matrix resulting from source masking as discussed in our Methods section. Therefore we show it as a comparison but do not use it in our modelling. The error bars are 1*σ* uncertainties that account for all sources of noise and error, including map-making, calibration, detector noise, and cosmic variance associated with finite size of the survey. (**b**) Optical and infrared diffuse galactic light (DGL) spectrum. The CANDELS points are taken from the DGL model components at 10^4^≤ℓ≤3 × 10^4^, and the CIBER points are taken directly from Fig. 2. of ref. 16 where they subtract off the shot noise component from their data. The galactic latitude for the optical points are |*b*|≃39°,32°,41°,40° for the points labelled Witt^55^, Paley^56^, Ienaka^57^ and Guhathakurta^58^. The Brandt^59^ points are modeled over the full sky. GOODS-S is at a galactic latitude of |*b*|**=**54^°^.

Our measurements continue to show the significant excess in the fluctuation amplitude at 30 arcsec and larger angular scales, when compared with the clustering of faint, low-redshift galaxies^20^. The large-scale fluctuations correlate between filters (Fig. 4). The excess in the amplitude of fluctuations relative to faint low-redshift galaxies is consistent with previous measurements at 3.6 μm (refs 13, 15). Our HST-based power spectra probe deeper into the fluctuations and have shapes departing from the fluctuations measured with the CIBER sounding rocket experiment^16^. Due to the shallow depth of the CIBER imaging data, the measured fluctuations there are dominated by the shot noise of the residual galaxies at the arcmin angular scales that we probe here with Hubble data (Fig. 5).

**Figure 6:**
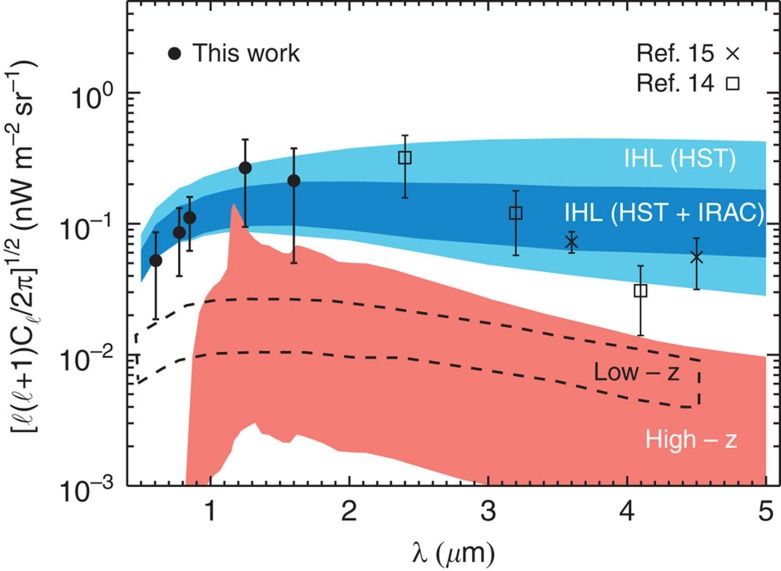
Spectral energy distribution of optical and infrared fluctuations at arcmin angular scale. The Hubble/CANDELS points are averaged over 10^4^≤ℓ≤3 × 10^4^, with the best-fit shot noise and DGL components subtracted. Our model fits for the high-redshift and IHL components, with their 1*σ* bounds, are shown as the filled regions. The errors here are propagated from the errors on the auto spectrum at the same ℓ range. The light blue region shows the 1*σ* confidence bound for the IHL component when we use only the HST data in our model fitting; the dark blue region shows the 1*σ* confidence bound for the IHL component when use both the HST and Spitzer IRAC data in our model fitting. The light red coloured region signifies the 1*σ* error bound for the high-redshift model component. The dashed line corresponds to the 1*σ* bound for the low-redshift component. The Spitzer^15^ and AKARI^14^ data are taken from previous measurements at ℓ=3,000. Note the spectral dependence difference between the high-redshift signal and IHL. Below 0.8 μm we do not expect any signal from *z***>**8 galaxies. The presence of fluctuations at optical wavelengths requires a low-redshift signal in addition to high-redshift sources to explain combined optical and infrared background intensity fluctuations.

At angular scales of tens of arcmin and above, CIBER detected an up-turn in the fluctuations with an amplitude well above the level expected from instrumental systematics and residual flat-field errors^16^. However, as shown in Fig. 5, the combination of CIBER and Hubble fluctuations is consistent with a power-law clustering signal out to the largest angular scales probed by CIBER. If the power-law signal is of the form *C*_ℓ_∝ℓ^*α*^, the best-fit slope to combined CIBER and Hubble measurements at 1.6 μm is *α* =−3.05±0.07. This slope is consistent with Galactic dust, which in emission at 100 μm has a power-law of −2.89±0.22 (ref. 27). At the largest angular scales we could be detecting interstellar light scattered off of Galactic dust or diffuse Galactic light (DGL). The overall amplitude of fluctuations we measure at 1.6 μm is consistent with 10% DGL fluctuations at tens of arcmin angular scales, given the 100 μm surface brightness of GOODS-S of ∼0.5 MJy/sr, and existing DGL intensity measurements (Fig. 6). Future measurements in the optical wavelengths over a wider area are necessary to confirm the Galactic nature of fluctuations at angular scales >1°.

### Multicomponent model

For our theoretical interpretation, we invoke a model which involves four main components: (a) intrahalo light (IHL) following ref. 15, (b) diffuse galactic light (DGL) due to interstellar dust-scattered light in our Galaxy, (c) low-redshift residual faint galaxies^20^; and (d) the high-redshift signal. We assume the flat ΛCDM model with Ω_M_**=**0.27, Ω_b_**=**0.046, *σ*_8_**=**0.81, *n*_s_**=**0.96 and *h***=**0.71 in our theoretical modelling^28^. We summarize the basic ingredients of our model now while providing references for further details.

For IHL, we follow the model developed in ref. 15. The mean luminosity of the IHL at rest-frame wavelength *λ* for a halo with mass *M* at *z* is described as


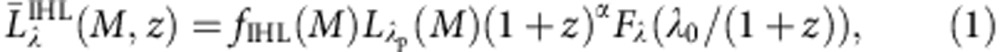


where *λ*_0_**=***λ*(1+*z*) is the observed wavelength, *α* is the power-law index which takes account of the redshift-evolution effect and *f*_IHL_(*M*) is the IHL luminosity fraction of the total halo, which takes the form


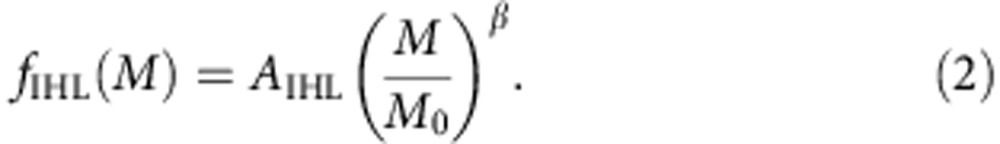


Here *A*_IHL_ is the amplitude factor, *M*_0_=10^12^ *M*_⊙_, and *β* is the mass power index. In equation (1), *L*_*λp*_(*M*)**=***L*_0_(*M*)/*λ*_*p*_ is the total halo luminosity at 2.2 μm and at *z***=**0, where *λ*_*p*_**=**2.2 μm and *L*_0_ is given by^29^


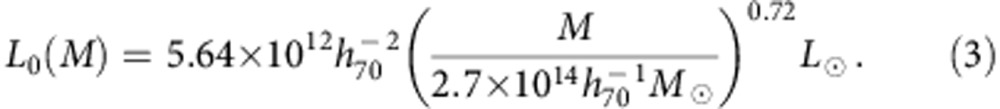


Here *H*_0_**=**70*h*_70_ km s^−1^ Mpc^−1^ is the present Hubble constant. The *F*_*λ*_ term is the IHL spectral energy distribution (SED), which can transfer 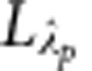 to the other wavelengths and is normalized to be 1 at 2.2 μm (see the discussion in ref. 15 for details). We assume the IHL SED to be the same as the SED of old elliptical galaxies, which are comprised of old red stars^30^.

The angular power spectrum of IHL fluctuations, 
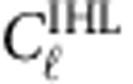
, is calculated via a halo model approach^31^ and involves both a one-halo term associated with spatial distribution inside a halo and a two-halo term involving clustering between halos. The details are provided in ref. 15. In the clustering calculation, we assume the IHL density profile follows the Navarro–Frenk–White (NFW) profile^15^,^32^. We set the maximum IHL redshift at *z*_m*ax*_**=**6. The *M*_min_ and *M*_max_ are fixed to be 10^9^ and 10^13^ M_⊙_* *h^−1^, and the power-law index *β* is fixed to be 0.1 in this work^15^. The IHL model is then described with two parameters: *A*_IHL_ in equation (2) and the power-law index *α* in equation (1).

A simple test of IHL is to grow the source mask and study how the fluctuation power spectrum varies as a function of the mask size. However, we note that our model for IHL involves clustering at large angular scales. That is, in our description IHL is not restricted to regions near galactic disks only. The dependence of the power spectrum with mask size is studied in refs 33, 34. These studies find that the fluctuations do not vary strongly with the mask radius, though such studies ignored the mode-coupling effects associated with the mask as the mask radius is varied. As discussed in ref. 15, to test IHL one has to grow the masking area to a factor of 10 larger than the typical mask radius used in the current analyses of fluctuations. In the case of Spitzer data, where we expect fluctuations to be dominated by IHL, the relatively large 2 arcsec point spread function makes it close to impossible to test IHL directly with a varying mask. However, additional tests of IHL exist in the literature^33^. These include correlations between artificial halos and masked sources, and correlations between masked sources and foreground galaxies. Such tests have not ruled out the IHL component. Furthermore, without such a component we are not able to explain the fluctuations measured at wavelengths below 0.8 μm, as residual fiant galaxy clustering^20^ is not adequate to explain the measurements.

The DGL component involves dust-scattered light and it is likely that the same dust is observed by IRAS at far-infrared wavelengths through thermal emission. The DGL component was considered in ref. 16 and an upper limit on the expected amplitude was included based on the cross-correlation with 100 μm IRAS map of the same fields. The CIBER final model results focussing on IHL to explain the fluctuations did not allow the DGL fluctuations amplitude to vary as a free parameter. With Hubble data, we find stronger evidence for DGL or a DGL-like signal once combined with CIBER, and with an (root mean squared) r.m.s. amplitude for fluctuations that is at least a factor of 3 larger than the upper limit used in ref. 16 based on the cross-correlation with CIBER. Moreover, we find that the angular power spectrum is proportional to ∼ℓ^−3^ over the degree scales measured from CIBER to tens of arcmin scales of CANDELS measurements. So we model it with an amplitude factor *A*_DGL_ as


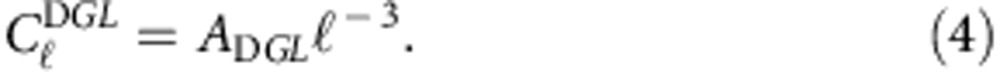


To validate this ℓ^−3^ DGL slope dependence, we perform a linear fit in log space at low multipoles of the HST 1.6-μm data simultaneously with the CIBER 1.6-μm data. We measure a slope of −3.05±0.07, so the functional form of our DGL model with ℓ^−3^ is appropriate (Fig. 5). The power-law behaviour of the DGL signal is consistent with Galactic dust emission power spectra in far-infrared and sub-mm surveys^27^, and dust polarization measurements in all-sky experiments like Planck^35^. We summarize a comparison of our DGL intensity measurements with those of existing measurements as a function of wavelength in Fig. 5.

The clustering of low-redshift faint galaxies at *z*<5, where reliable luminosity functions exist in the literature, is based on the detailed models in ref. 20. We follow the calculations presented there to establish the expected level of low-redshift clustering, and the uncertainty of that expectation at the depth to which we have masked foreground galaxies. Because the low-redshift luminosity functions are not steep, unless there is a break or steepening in the luminosity function—which is not supported by the halo model—these low-redshift populations do not dominate the clustering we have measured. Given that our fluctuation measurements reach the deepest depths provided by both Hubble and Spitzer/IRAC, it is also unlikely that populations such as extreme red galaxies at *z*<2 are responsible for the measured fluctuations. If there are populations at low redshift responsible for the SED of the fluctuations we measure, then they would need to have individual SEDs that are consistent with a sharp break redshifted between 0.8 and 1.25 μm. While fluctuation measurements in just two bands cannot separate galaxies that have redshifted 4,000-Å break, or galaxies that have red-shifted Lyman-*α* break between those two bands, with five bands we have adequate knowledge on the SED of fluctuations, and the shape of the clustering over two decades in angular scales to separate high-*z* galaxies from low-*z* faint interlopers. The low-*z* interlopers are also likely captured by our IHL model as we cannot distinguish between diffuse stars and faint, dwarf galaxies that happen to be a satellite of a large dark matter halo in our modelling description.

### Galaxies during reionization

The final and critical component in our model is the signal from *z***>**6. We break this signal into two redshift intervals given the placement of the five ACS and WFC3 bands, based on the Lyman-dropout signal that moves across these bands. In particular, we consider 8<*z*<13 and 6<*z*<8 as the two windows. As we discuss later, given the availability of SFR density measurements in the 6<*z*<8 interval, we mostly allow the signal in that redshift interval to be constrained by the existing data, and model-fit independently the SFR density in the higher redshift interval. We do not have a strong independent constraint on the 6<*z*<8 signal since it is only a Lyman-dropout in our shortest-wavelength band at 0.6 μm. This allows a better separation of the 8<*z*<13 from the rest of the signals discussed above. To measure 6<*z*<8 independently, we would need at least one more band below 0.6 μm. The signal from 8<*z*<15 disappears from the three optical bands (0.6–0.85 μm) and is present in the two IR bands at 1.25 and 1.6 μm.

To model the high-redshift signals, we adopt an analytic model^19^,^36^ based on the work of ref. 37. It involves a combination of two separate classifications of stars—moderate-metallicity, second-generation or later stars (PopII) and the first generation of stars ever formed in the Universe, hence zero metallicity (PopIII). These are modelled with Salpeter^38^ and Larson^39^ initial mass functions (IMFs) for PopII and PopIII stars, respectively. The calculation related to direct stellar emission and the associated nebular lines, including especially Lyman-*α* emission, follows the work of Fernandez and Komatsu^37^. The total integrated intensity from *z*_min_<*z*<*z*_max_ is


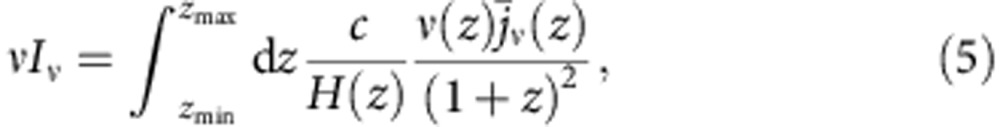


where *ν*(*z*)**=**(1+*z*)*ν*. The comoving specific emissivity, as a function of the frequency is composed of both PopII and PopIII stars with an assumed *z*-dependent fraction as discussed in ref. 36 with the form given by


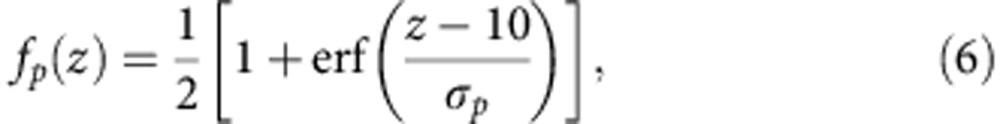


with *σ*_*p*_**=**0.5. The model thus assumes most of the halos have PopIII stars at *z***>**10, while PopII stars dominate at redshifts lower than that.

There are a number of theoretical parameters related to this model, especially the escape fraction of the Lyman-*α* photons *f*_esc_, the star-formation efficiency denoting the fraction of the baryons converted to stars in high-redshift dark matter halos, or f_*_, and the minimum halo mass to host galaxies, or *M*_min_. The overall quality of the data is such that we are not able to independently constrain all of the parameters related to the high-redshift intensity fluctuation signal. Moreover these parameters are degenerate with each other (that is, changing f_*_ can be compensated by a change in *M*_min_, for example). Thus given that we do not have the ability to constrain multiple parameters, we simply model-fit a single parameter *A*_high−z_ that scales the overall amplitude from the default model, interpret that scaling through a variation in f_*_, and subsequently convert that to a constraint on the SFRD. We fix our default model to a basic set of parameters, and assume *f*_esc_**=**0.2, M_min_**=**5 × 10^7^ M_⊙_, and f_*_=0.03. The resulting optical depth to reionization of this default model is 0.07, consistent with the optical depth measured by Planck^8^. Among all these parameters, the most significant change (over the angular scales on which we measure the fluctuations) comes effectively from f_*_, or the overall normalization of 
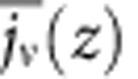
, given that it is directly proportional to f_*_. This can in turn be translated to a direct constraint on the SFRD, *ψ*(*z*), since with f_*_ we are measuring the integral of the halo mass function such that


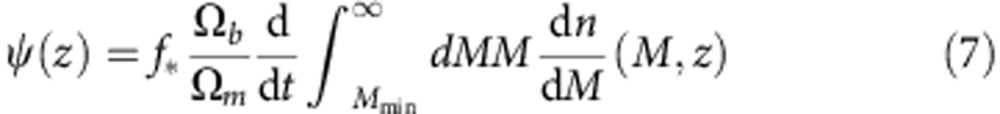


where d*n*/d*M* is the halo mass function^40^.

Finally, to calculate the angular power spectrum of fluctuations, we also need to assign galaxies and satellites to dark matter halos. For that we make use of the halo model^31^. We make use of the same occupation number distribution as in ref. 36 where the central and satellite galaxies are defined following ref. 41. However, departing from the low-redshift galaxy models, we take a steep slope for the satellite counts in galaxies with *α*_*s*_**=**1.5. The low-redshift galaxy clustering and luminosity functions are consistent with *α*_*s*_∼1 (ref. 41), but such a value does not reproduce the steep faint-end slopes of the Lyman-break galaxy (LBG) luminosity functions^3^,^4^,^5^,^6^,^7^. Such a high slope for the satellites also boost the non-linear clustering or the 1-halo term of the fluctuations. We do not have the ability to independently constrain the slope of satellites from our fluctuation measurements. In the future a joint analysis of fluctuations and LBG luminosity functions may provide additional information on the parameters of the galaxy distribution that is responsible for fluctuations. It may also be that the models can be improved with additional external information, such as the optical depth to reionization. We also note that other sources at high redshift include direct collapse black holes (DCBHs^19^), but we do not explicitly account for them here as the existing DCBH model is finely tuned to match Spitzer fluctuations, and the low signal-to-noise ratio of the Chandra–Spitzer cross-correlation results in them residing primarily at *z***>**12. DCBHs at such high redshifts will not contribute to Hubble fluctuations.

Finally, at smaller angular scales, the shot noise dominates the optical and infrared background intensity fluctuation. Since it is scale independent, we set it as a free variable parameterized as


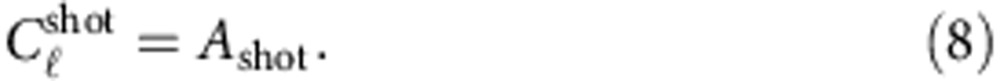


This noise term in the fluctuation power spectrum arises because of the Poisson behaviour of the galaxies at small angular scales, a product of the finite number of galaxies. Our measured shot noise comes from a combination of the unmasked, faint low-redshift dwarf galaxies and the high-redshift population. We do not use the information related to the shot noise in our models, but instead treat it as a free independent parameter, since we cannot separate the high-redshift shot noise from the shot noise produced by faint, low-redshift dwarf galaxies. Here we focus mainly on the clustering at tens of arcseconds and larger angular scales to constrain SFRD during reionization. In the future, with either a precise model for the low-redshift galaxies or a model for high-redshift galaxies that determines their expected number counts as a function of the free parameters such as *M*_m*in*_ and f_*_, it may be possible to separate the overall shot noise associated with reionization sources from that of the low-redshift faint galaxies. If this is the case, then it might also be possible to improve the overall constraints on the high-redshift population. It may also be that under an improved model, shot noise may end up providing complementary information to galaxy clustering to break certain degeneracies in model parameters.

Our overall model for the optical and infrared background fluctuations is


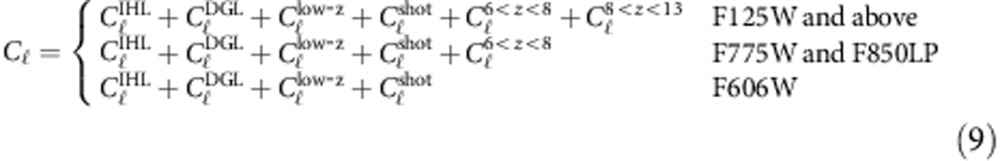


Given that we are not able to constrain the amplitude of 
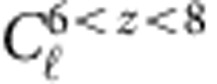
 given the degeneracies with the parameters involving the IHL model, and the fact that we only have a single band below it, we set 
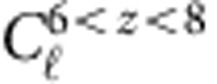
 based on the default prediction of our model, but allow the overall amplitude *A*_6<*z*<8_ to vary such that it uniformly samples the SFRD between [0.003,0.2] M_⊙_ yr^−1^ Mpc^−3^. The range is fully consistent with the existing measurements on the SFRD between *z***=**6 and 8 (refs 3, 4, 5, 6). Our constraint on *A*_8<*z*<13_ is mostly independent of this parameter since we can safely constrain the Lyman-dropout signal between 0.8 and 1.25 μm with our existing data.

We also included the CIBER^16^ data at 1.1 and 1.6 μm and Spitzer^15^ data at 3.6 μm in our fitting process. When compared with the Hubble data at 1.25 and 1.6 μm, we find the CIBER data are likely dominated by the emission from a DGL-like signal at large angular scales, and low-*z* faint galaxies at *z*<5 at small angular scales (Fig. 5). For the fluctuations from faint, low-*z* galaxies, we adopt a model of residual galaxies that is derived from the observations of the luminosity function for different near-infrared bands^20^. This model already includes the shot-noise term, and we add a scale factor *f*_low-*z*_ to vary the low-*z* angular power spectrum, 
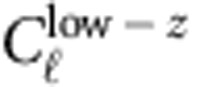
, in 1*σ* uncertainty. For the DGL component, we use the 
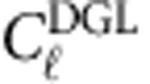
 of Hubble data at 1.25 and 1.6 μm to fit the CIBER data at 1.1 and 1.6 μm.

We perform joint fits for Hubble, CIBER and Spitzer data with the Markov Chain Monte Carlo (MCMC) method. The Metropolis–Hastings algorithm is used to find the probability of acceptance of a new MCMC chain point^42^,^43^. We estimate the likelihood function as 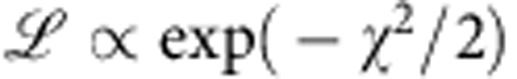 , where *χ*^2^ is given by


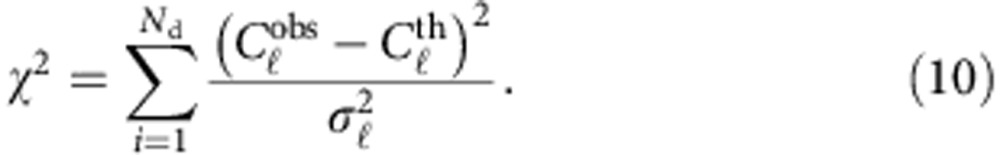


Here 
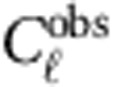
 and 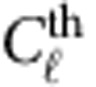 are the observed and theoretical angular power spectra for HST, Spitzer or CIBER data, respectively. *σ*_ℓ_ is the error for each data point at ℓ, and *N*_d_ is the number of data points. The total *χ*^2^ of HST, Spitzer and CIBER is 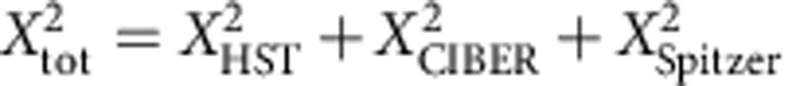.

**Figure 7:**
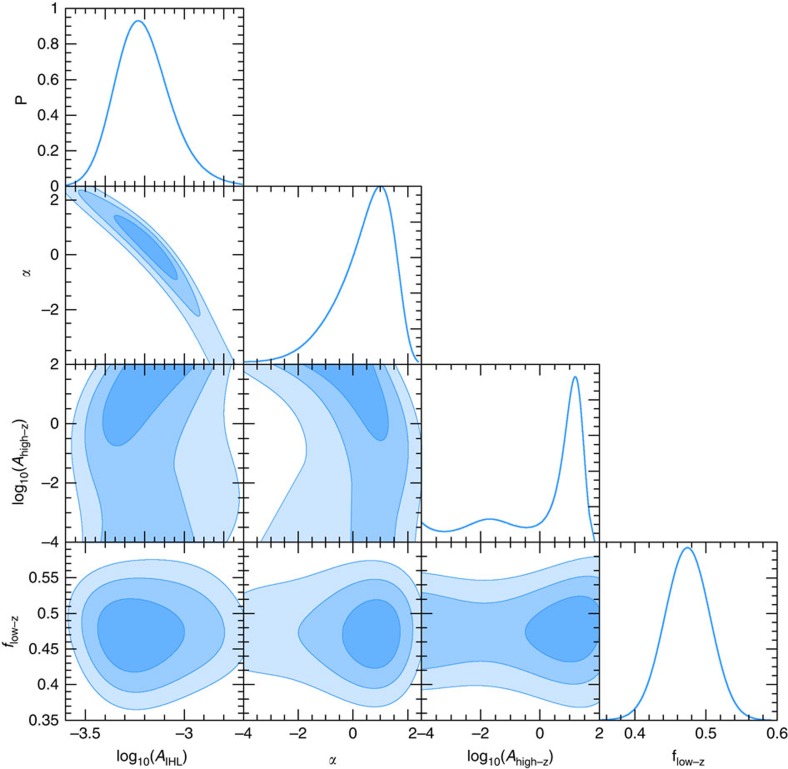
Probability distributions of fitted model parameters. Here we show the probablity density distributions for our fitted model parameters log_10_(A_IHL_), *α*, *f*_low-z_, and log_10_(A_high-z_) corresponding to the distribution from 8≤*z*≤13. The single curves on the outermost column of each row, labelled with a ‘P', show the marginalized probability distribution for each parameter labelled on the bottom of the figure. Contour regions to the left of these probability distributions show how the parameters scale with one another. Each of the shaded regions in the contours correspond to the 1, 2 and 3*σ* uncertainty ranges.

We assume a flat prior probability distribution for the free parameters; see Table 1 for prior information. Both *A*_DGL_, 
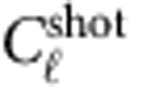
 vary as independent parameters for each band. Both the *A*_DGL_ and 
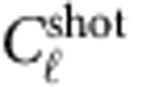
 parameters are sixfold, with one for each HST band and for Spitzer/IRAC 3.6 μm. (we combined the two CIBER bands with two of the HST bands). We have two parameters for IHL and one parameter for the normalization of the reionization galaxies with *A*_8<*z*<13_. We have two more parameters that we vary, *A*_6<*z*<8_ and *f*_low−*z*_. We set a uniform prior on *A*_6<*z*<8_ in the SFRD following the existing measurements to be between 0.003 and 0.2 M_⊙ _yr^−1^ Mpc^−3^. We also set a uniform prior on *f*_low-*z*_ over a reasonable range of models to account for the overall uncertainty in the models of ref. 20 to describe the *z*<5 faint galaxy clustering at the same masking depth as our measurements. We marginalize over both *A*_6<*z*<8_ and *f*_low-*z*_ as well as all other parameters when quoting results for an individual parameter. We have a total of 14 free parameters in our MCMC fitting procedure that we extract from the data. Among these parameters, 12 of them simply describe the small and large angular scale fluctuations in each of the bands we have performed the measurements. These parameters are summarized in Table 1. We generate twenty MCMC chains, where each chain contains about 100,000 points after convergence. After thinning the chains, we merge all chains and collect about 10,000 points for illustrating the probability distributions of the parameters. Contour maps for each of the fitted model parameters are shown in Fig. 7. Our best-fit model with 14 free parameters have a minimum *χ*^2^ value of 278 for a total degrees of freedom of *N*_dof_**=**104.

## Discussion

**Figure 8:**
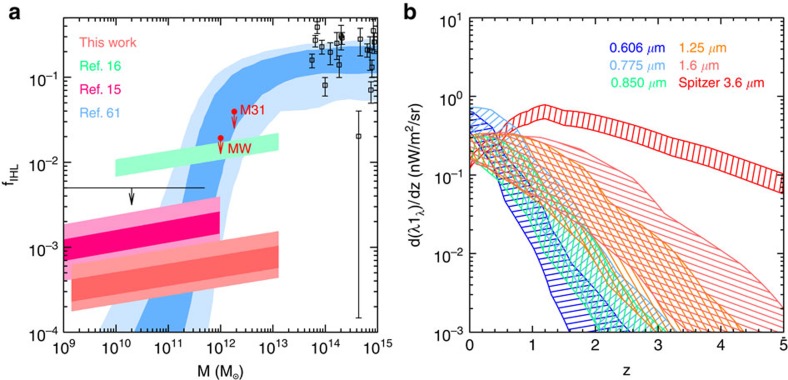
Intrahalo light fraction and model intensities. (**a**) *f*_IHL_, the intrahalo light fraction, as a function of halo mass. The dark and light shaded regions show the 95 and 68% ranges of *f*_IHL_ from anisotropy measurements, and from an analytical prediction^60^ (blue). Intracluster measurements are shown as boxes^61^, with 1*σ* errors. The red downward arrows denote the 95% confidence upper limit on *f*_IHL_ estimated for Andromeda (M31) and our Milky Way (MW), following Fig. 2 of ref. 15. (**b**) *d*(*λI*_*λ*_)/*dz* from the model, as a function of redshift. We show the 68% confidence uncertainties derived from MCMC fitting of the data at 0.606, 0.775, 0.850, 1.25 and 1.6 μm. The total IHL intensity is 
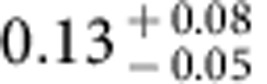
,
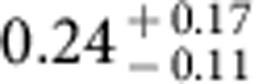
,
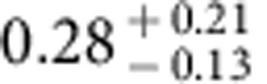
,
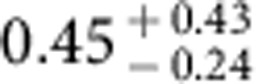
, and 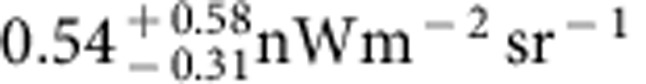 for 0.606, 0.775, 0.850, 1.25 and 1.6 μm, respectively.

Our results are summarized in Fig. 3, where we show the best-fit model curves. While the dominant contribution to the excess fluctuations comes from DGL at ℓ<10^4^, at intermediate scales we find the IHL and reionization contributions to be roughly comparable. In Fig. 6 we show the r.m.s. fluctuation amplitude at ∼5 arcmin angular scales over the interval 10,000<ℓ<30.000. We find a spectral energy distribution that is consistent with Rayleigh–Jeans from 4.5 to 2.4 μm, but diverges between 2.4 and 1.6 μm, and even more rapidly between 1.25 and 0.85 μm. The fluctuations can be explained with a combination of IHL and high-redshift galaxies. The residual low-z galaxy signal is small but non-negligible. We find that it is mostly degenerate with IHL, especially if we allow its amplitude to vary more freely than the range allowed by the existing models based on *z*<5 galaxy luminosity functions^20^. Thus, modelling uncertainities related to the low-*z* galaxy confusion do not contaminate our statements about reionization. Assuming the existing low-*z* galaxy model^20^, the best-fit model is such that the IHL intensity peaks at lower redshifts with decreasing wavelength (Fig. 8). At 3.6 μm, the IHL signal is associated with galaxies at *z*∼1, while at 0.6 μm over 80% of the signal is associated with galaxies at *z*<0.5. The total intensities are 
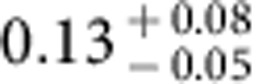
, 
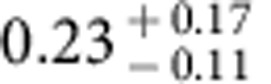
, 
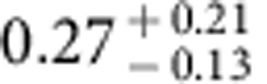
, 
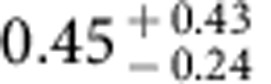
 and 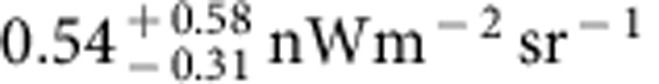 at 0.60, 0.77, 0.85, 1.25 and 1.6 μm, respectively. We find that the implied IHL intensities at 1.25 and 1.6 μm are a factor of 10 lower than the implied IHL intensities for a model of CIBER fluctuations with IHL alone. The difference is due to the CIBER model that only included IHL and ignored the presence of DGL.

The drop in the fluctuation amplitude from 1.25/1.6 μm to 0.85 μm allows for a signal from reionization, but the presence of fluctuations at shorter wavelengths, such as 0.6 μm, rules out a scenario in which reionization sources are the sole explanation for the fluctuations at wavelengths at 1 μm and above. The 3.6 μm and X-ray cross-correlation^18^ was explained with primordial direct collapse blackholes at *z***>**12 (ref. 19). In our multicomponent model we are able to account for the presence of fluctuations at short wavelengths with IHL, DGL and faint low-redshift galaxies, while a combination of those components and high-redshift galaxies are preferred to account for fluctuations at 1.25 and 1.6 μm. The high-*z* signal is modelled following the calculations in ref. 36. The signal has an overall amplitude scaling that is related to the star-formation rate during reionization. The bright end of the counts are normalized to existing luminosity function measurements, and the faint-end of the luminosity functions to have a steeper slope than measured with counts extending down to arbitrarily low luminosities. To test whether a component at high redshift is required to explain the measurements, we also re-ran the MCMC model fits but with *A*_high-*z*_ fixed at 0. In this case our best-fit model with 13 free parameters has a minimum *χ*^2^ value of 283 for a total degrees of freedom of *N*_dof_**=**105. The difference in the best-fit *χ*^2^ values with and without a model for high-redshift galaxies suggests a *P* value of 0.025. This is consistent with the 2*σ* to 3*σ* detection of 8<*z*<13 signal in the fluctuations (Fig. 6).

With multiwavelength measurements extending down to the optical, we are now able to constrain the amplitude of that signal with a model that also accounts for low-redshift sources in a consistent manner. This improves over previous qualitative arguments that have been made, or models involving high-redshift sources alone that have been presented, for the presence of a signal from reionization in the infrared background fluctuations^13^,^14^,^19^. In our models, the total intensity arising from all galaxies at *z***>**6 is log *νI*_*ν*_=−0.32±0.12 in units of nW m^−2^ sr^−1^ at 1.6 μm. At 1.6 μm the intensity from high-redshift sources is dominated by *z***>**8 galaxies, while at 0.85 μm we find an intensity log*νI*_*ν*_=−0.75±0.05 in units of nW m^−2^ sr^−1^ for 6<*z*<8 galaxies. The total intensity from *z***>**8 galaxies in the 1.6-μm band is comparable to the IHL intensity at the same wavelength (Fig. 8). However, at 3.6 μm, the IHL signal is a factor of about five times brighter than the *z***>**8 galaxies. At 1.6 μm the total of the IHL, high-*z*, and integrated galaxy light^44^ of 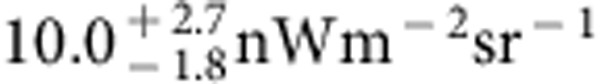 is comparable to the EBL intensity inferred by gamma-ray absorption data^45^ of 15±2(stat)±3(sys).

**Figure 9:**
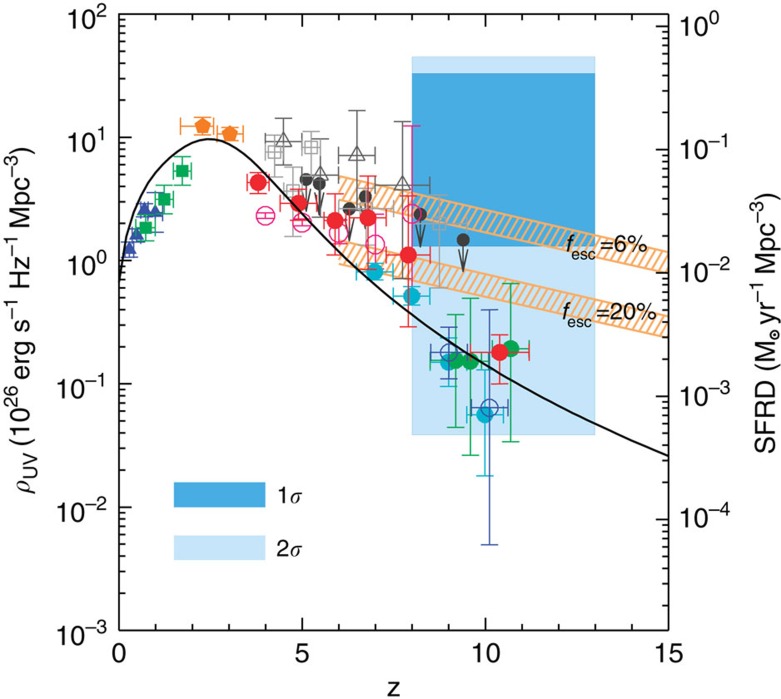
The ultraviolet luminosity density and star-formation rate density as measured with intensity fluctuations. Plotted here is the specific ultraviolet luminosity density (left axis), with the equivalent star formation rate density (SFRD, right axis), as a function of the redshift *z*. We show the 1*σ* and 2*σ* error bounds in our redshift bin as the light and dark blue regions. Results from low-redshift surveys are shown as blue triangles^62^, bright green squares^63^, and orange pentagons^64^. At *z*∼4 to 10 the star formation rate density is shown to decrease with increasing redshift as measured by previous works, plotted as filled cyan circles^65^, filled red circles^6^, open red circles^7^ filled green circles^4^,^5^ and open blue circles^3^. Gamma ray burst (GRB) studies are shown as grey triangles^66^, squares^67^ and dark grey circles^68^. Except for ref. 65, other estimates are luminosity function extrapolations and integrations down to *M*_UV_=−13. Our measured star formation rate densities are consistent with previous works between *z*∼8 to 10, however only extremely bright galaxies are directly detected in the aforementioned works with extrapolations down to *M*_UV_=−13 involves the measured faint-end slope of the luminosity function. For reference we plot the theoretically expected relation^69^ between ultraviolet luminosity density and redshift to reionize the universe and/or to maintain reionization using an optical depth to reionization of *τ***=**0.066±0.012 (ref. 8). We take a gas clumping factor of *C***=**3 and show two cases where the escape fraction of galaxies *f*_e*sc*_ is 6 and 20% (see also ref. 47).

Using the best-fit model and uncertainties as determined by MCMC model fits, we also convert the *A*_8<*z*<13_ constraint to a measure of the luminosity density of the universe at *z***>**8 (Fig. 9). The resulting constraint is 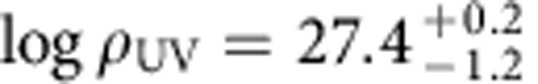 in units of erg s^−1^ Hz^−1^ Mpc^−3^ at (1*σ*). As shown in Fig. 9, the 68% confidence level constraint on *ρ*_UV_ is higher than the existing results from Lyman drop-out galaxy surveys during reionization at *z***>**8 (refs 46, 47), and especially at *z*∼10 (ref. 4). At the 95% confidence level, our measurement is fully consistent with the existing results at *z*∼10 (ref. 48). Our constraint allows for the possibility that a substantial fraction of the ultraviolet photons from the reionization era is coming from fainter sources at depths well below the detection threshold of existing Lyman-dropout surveys, as is indeed anticipated from the steep measured slopes of the ultraviolet luminosity functions from detected galaxies. Despite their lack of detections in the deepest surveys with HST, the majority of the faint sources responsible for both fluctuations and reionization should be detectable in deep surveys with JWST centred at 1 μm.

## Methods

### Field selection

To obtain angular power spectra over large angular scales, individual exposures must be combined into one or more mosaiced images. We generate our own mosaics using the self-calibration technique^25^ (SelfCal), instead of using the publicly available mosaiced images produced by astrodrizzle (available at http://candels.ucolick.org/data_access/Latest_Release.html). Foreground emissions, predominately that of Zodiacal light, are particularly pernicious at infrared wavelengths^49^, so care must be taken when producing mosaics which combine observations taken at different times, especially at the WFC3/IR channels. Offsets between frames will lead to a fictitious anisotropy signal if care is not taken to properly model and remove those offsets, which is what SelfCal was designed to do.

Although the CANDELS observations cover multiple fields, only the two deep fields−the Great Observatories Origins Deep Survey-South and -North (GOODS-S and GOODS-N)^23^—have sufficient overlap between frames to perform a self-calibration. The GOODS-N data set has a larger number of frames with clear overall offsets resulting from scattered light than does GOODS-S. Therefore we have restricted our analysis to GOODS-S, which has an area of approximately 120 square arcmins. The wider CANDELS fields are composed of much poorer tile patterns, as can be seen in Fig. 18 of ref. 21, which are a significant drawback for fluctuation studies, since one cannot calibrate the full mosaic to a consistent background level without introducing artificial gradients to the background intensity. Such dithering patterns were pursued by CANDELS to maximize the total area covered with WFC3. The increase in area is of benefit to studies that aim to detect rare galaxies, such as LBGs at *z***>**5.

### Initial data reduction and map making

In addition to the data collected by CANDELS, the GOODS-S field has a wealth of HST archive data, publicly available on the Barbara A. Mikulski Archive for Space Telescopes (MAST; located at https://archive.stsci.edu/hst/search.php). We assembled our own collection of calibrated, FLT frames from the MAST archive, comprised of some or all of the data from ten different HST proposals^21^,^22^,^23^,^50^. These data are also supplemented by the Early Release Science observations^24^. The tile patterns of these observations can be found in Fig. 1.

In addition to selecting frames with a favourable tile pattern appropriate for self-calibration, we also had to take two additional potential issues into account. After the replacement of the ACS CCD Electronics Box during the fourth Hubble servicing mission (SM4), ACS imaging data are plagued with horizontal striping dominated by 1/*f* noise. Furthermore, ACS frames have a tendency to introduce a Moiré pattern (correlated noise) when the pixel scale is modified in a low signal-to-noise area. Both of these characteristics can potentially contaminate an angular power spectrum to such an extent that the systematics dominate the measurement. With simulations, we found that with an increased number of ACS frames taken with varying position angles effectively removes the Moiré pattern upon repixelization, and the bias-striping issue is ameliorated by simply omitting a large percentage of post-SM4 frames. Any given collection of ACS frames we used contained <27% post-SM4 frames.

Our MAST archive data were initially reduced with PyRAF version 2.1.1. MAST queries are reprocessed ‘on-the-fly', which entails using the most recent calibration files. Thus the FLT frames we retrieved from the archive had standard calibrations of bias and dark frame subtraction, along with flat-field correction, already performed. We identified cosmic rays in the the FLT frames with the CRCLEAN PyRAF module; sub-arcsecond astrometric alignment against the publicly available CANDELS mosaics(http://candels.ucolick.org/data_access/GOODS-S.html) was achieved with tweakreg. All ACS data were charge transfer efficiency corrected, and post-SM4 ACS frames were destriped prior to charge transfer efficiency correction.

We generate mosaics from the reduced FLT frames using the same SelfCal model as in ref. 51, an example where HST data has already been self-calibrated; details of the model can be found there. We deweight bad pixels and cosmic rays, and iterate three times to find a SelfCal solution. Our input FLT frames are geometrically distorted with a pixel size of 0.′′0498 × 0.′′0502 for ACS, and 0.′′1354 × 0.′′1210 for WFC3. We remove the distortion in the map making procedure and produce mosaics with a slightly larger pixel size of 0.′′140 (geometrically square).

Note that the algorithm we use is same as the one used to generate self-calibrated maps of IRAC in the Spitzer fluctuation studies^13^,^15^,^52^. The same method was implemented by the Herschel SPIRE Instrument Science team to generate wide area mosaics of the Herschel-SPIRE data, resulting in far-infrared fluctuations^27^. The algorithm originates from the time of FIRAS^25^ and has wide applications. In the future we expect it will be used to combine frames and produce stable wide-area mosaics from JWST, Euclid, and WFIRST, among others.

We generate two maps per band so we can use the differences and sums to study systematics and noise biases, as was done in previous studies^15^. The data are sorted by observation date and every other FLT frame was used for each half map. HST data generally have two or more exposures per pointing, so this results in two maps per band of the same or similar dither pattern and exposure time per pointing. One map from each band can be found in Fig. 2. Multiple maps of the same band enable us to do cross-correlations, which ensures a removal of uncorrelated noise in the auto-spectra. This jack-knife process is similar to all other analyses related to large-scale structure and CMB angular power spectra from maps.

### Generation of resolved source mask

We utilized existing multi-wavelength catalogues of detected sources from the ultraviolet to mid-infrared (CITO/MOSAIC, VLT/VIMOS, VLT/ISAAC, VLT/HAWK-I, and *Spitzer*/IRAC)^26^. In addition, all the sources from the CANDELS, HUDF and Early Release Science surveys (F435W, F606W, F775W, F814W, F850LP, F098M, F105W, F125W, and F160W) are also present in the catalogue. The 50% completeness limit for F160W in the catalogue is *m*_AB_**=**25.9,26.6 and 28.1 for the CANDELS wide, deep and HUDF regions respectively; the 5*σ* limiting magnitudes are 27.4, 28.2 and 29.7. For each source detected by any of the aforementioned instruments, we apply an elliptical mask with parameters corresponding to the SEXTRACTOR Kron elliptical aperture. This catalogue simplifies our masking procedure and ensures we are masking sources detected at other wavelengths, which may otherwise be undetected in our five bands.

In addition to the source mask generated from sources detected by other instruments, we also generate our own internal masks. We run SEXTRACTOR on a coadded map in each of our five filters and apply the same elliptical masking procedure to incorporate the shapes of sources. Next, we take the union of all five internal source masks, plus the source mask we made from the pre-existing catalogue. After applying this union mask to each band, we clip 5*σ* outliers and visually inspect each masked map. Any residual sources are masked by hand. We verified that all sources detected above 5*σ* in any of the bands, including deep IRAC data at 3.6 μm, are masked. This process yields 53% of the pixels unmasked for the Fast Fourier Transform (FFT) computation. Note that tests can be performed which expand and shrink the source mask to further test the IHL model (ref. 33).

### Absolute flux calibration

SelfCal achieves relative calibration between frames, so in general, the absolute flux calibration, or gain, of SelfCal output maps needs to be determined from a standard flux reference. The multi-wavelength catalogue used in the masking procedure is in principle a good enough reference, however, the photometry in the public CANDELS source catalogues were obtained with a private version of SExtractor, and has aperture corrections applied to the flux densities which were extracted from PSF-matched maps. We instead generate internal catalogues from public CANDELS MultiDrizzled mosaics using the same procedure as we used on our mosaics.

In each band, we repixelize the MultiDrizzle maps to our SelfCal pixel scale, and perform source extraction with SEXTRACTOR on both our mosaics and the MultiDrizzle mosaics. We use the same parameter files for all the source extractions. We then astrometrically match the resultant catalogues in each band and keep those sources that are common within a radius of 0′′.1 (our FLT frames were aligned in tweakreg with MultiDrizzle mosaics as the astrometric reference, so our astrometry is similar to the MultiDrizzle maps at sub-arcsecond scales). Counts in electron per second are converted to μJy using the current HST magnitude zero points^22^. The calibration introduces a 4–5% error which is propagated into our final error bars.

### Power spectrum evaluation

All the statistical information contained in any one of our maps is summarized by its angular power spectrum, *C*_ℓ_, which is just the variance of the 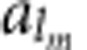
's. We used standard FFT techniques to estimate the *C*_ℓ_'s of our masked maps^13^,^15^,^16^,^27^. The mosaics can be seen in Fig. 2, both in real space and Fourier space. For each band we have two half maps, A and B. To measure the inherent noise in the data, we compute the noise power spectrum as the auto power spectrum of (A−B)/2. To measure the raw auto-spectrum, we compute the cross spectrum of the two half maps, A × B, which eliminates any uncorrelated noise in our power spectrum estimate. In general, the standard deviation at each multipole is


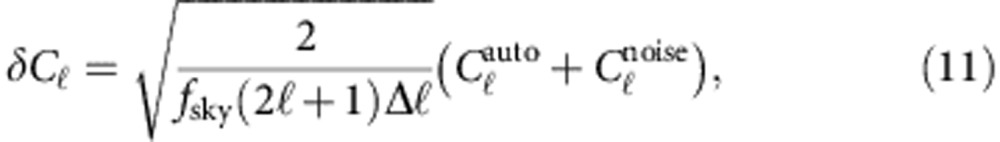


where Δℓ is the bin width for the given *C*_ℓ_, and *f*_*sky*_ is the fractional sky coverage from all the pixels used in the FFT (excluding zeros). In our case, we have some error associated with the absolute calibration of the maps, so we take the total error budget of our raw power spectra as the quadratic sum of the calibration errors with the variance in equation (1). The final measured auto-spectra and associated errors can be found in Supplementary Table 1.

To account for the effects that the source mask, tile pattern and finite beam size introduce into the power spectrum, we employ the correction techniques of the MASTER algorithm^53^, and closely follow the implementation procedures explained in Section 4, 5 and 6 of the Supplementary Information of ref. 15 (including Supplementary Information Fig. 1 of ref. 15). Among the procedures listed there, we have only slightly modified the way we generate our transfer function, T(ℓ). In addition to adding instrumental noise (step 2 in Section 6 of the Supplementary Information of ref. 15), we also add an offset to each tile equal to the median of the given FLT frame. This additional step should in principle be a good indicator of how well SelfCal is performing in offset removal, unique to each observation. Transfer functions for each of our five bands can be found in Supplementary Figure 2; measurements of the beam transfer function can also be found in Supplementary Figure 2.

## 

## Additional information

**How to cite this article:** Mitchell-Wynne, K. *et al*. Ultraviolet luminosity density of the universe during the epoch of reionization. *Nat. Commun*. 6:7945 doi: 10.1038/ncomms8945 (2015).

## Supplementary Material

Supplementary Information Supplementary Figures 1-2, Supplementary Table 1 and Supplementary Note 1

## Figures and Tables

**Table 1:** Proposal ID's for each filter.

Parameter	Best fit	Best fit (no high-*z*)	Prior min, max
log_10_(*A*_8≤*z*≤13_)	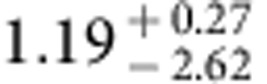	—	−5, 7
log_10_(*A*_*IHL*_)	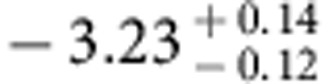	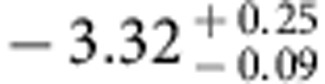	−6, 10
*α*	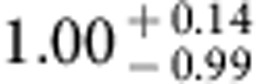	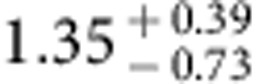	−5, 5
*f*_low−*z*_	0.47±0.03	0.47±0.03	0.1, 10
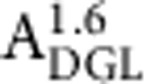	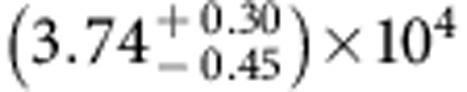	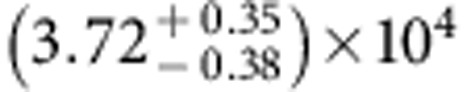	10^3^, 10^5^
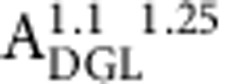	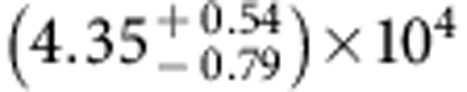	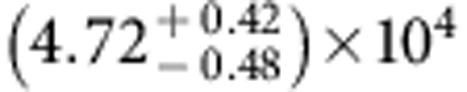	10^3^,10^5^
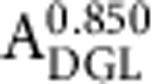	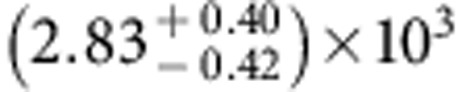	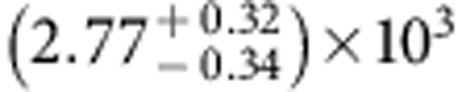	10^2^,10^4^
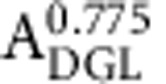	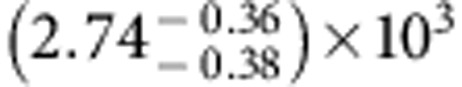	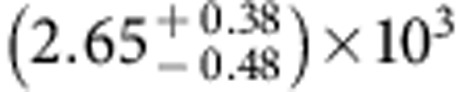	10^2^,10^4^
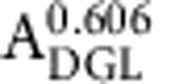	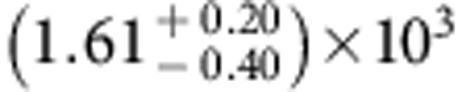	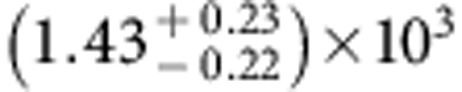	10^2^, 10^4^
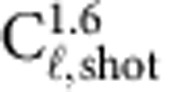	(7.54±0.13) × 10^−11^	(7.54±0.13) × 10^−11^	10^−11^,10^−10^
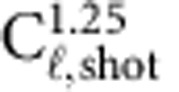	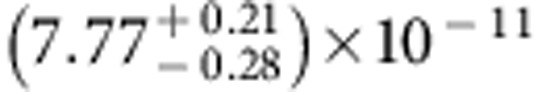	(7.77±0.14) × 10^−11^	10^−11^, 10^−10^
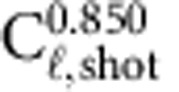	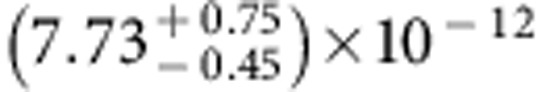	(8.10±0.45) × 10^−12^	10^−12^,10^−11^
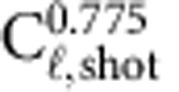	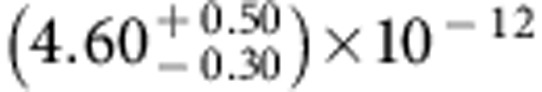	(4.65±0.30) × 10^−12^	10^−12^, 10^−11^
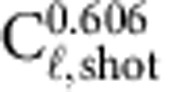	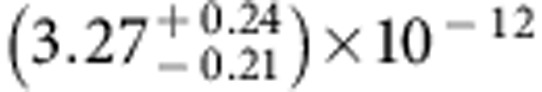	(3.39±0.15) × 10^−12^	10^−13^, 10^−11^

Filter	Proposal	IDs							
F606W	9500	9978	11563	12007	12060	12062	12099	12461	12534
WFC3	11359	12060	12061	12062					
ACS	9425	9803	10189	10258					
F850LP	9500	9978	10086						
F775W	9575								

Summary of free model parameters. The best-fit values are quoted with 1*σ* errors. log_10_(*A*_8≤*z*≤13_) is the high-redshift component used to constrain the SFRD during the reionization epoch, which is fit to the 1.25 and 1.60-μm bands. l*og*_10_(*A*_IHL_) and *α* are the two parameters necessary to describe the IHL component, 
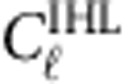
, in equation 9. *f*_low-*z*_ is the low-redshift scaling factor which varies the low redshift power spectrum within a 1*σ* uncertainty. *A*_DGL_^*i*^ and 
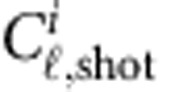
 are, respectively, the DGL amplitude scaling factor and shot noise at wavelength *i*. All parameter values have units of (nW m^−2^ sr^−1^)^2^. The best-fit model where l*og*_10_(*A*_8≤*z*≤13_) is non-zero with 14 free parameters (second column) have a minimum *χ*^2^-value of 278 for a total degrees of freedom of *N*_dof_**=**104. The case where l*og*_10_(*A*_8≤*z*≤13_)**=**0 with 13 free parameters (third column) has a minimum *χ*^2^-value of 283 for a total degrees of freedom of *N*_dof_**=**105. The ACS and WFC3 rows show the proposals that are common between all the bands in each instrument. For each proposal we did not necessarily use all the frames, specifically those from deep surveys.

## References

[b1] Loeb A. & Barkana R. The reionization of the universe by the first stars and quasars. Ann. Rev. Astron. Astrophys. 39, 19–66 (2001).

[b2] Fan X., Carilli C. L. & Keating B. Observational constraints on cosmic reionization. Ann. Rev. Astron. Astrophys. 44, 415–462 (2006).

[b3] Oesch P. A. . The most luminous z∼ 9-10 galaxy candidates yet found: the luminosity function, cosmic star-formation rate, and the first mass density estimate at 500 Myr. Astrophys. J. 786, 108 (2014).

[b4] Zheng W. . A magnified young galaxy from about 500 million years after the Big Bang. Nature 489, 406–408 (2012). 22996554 10.1038/nature11446

[b5] Coe D. . CLASH: Three Strongly Lensed Images of a Candidate z 11 Galaxy. Astrophys. J. 762, 32 (2013).

[b6] Bouwens R. J. . A census of star-forming galaxies in the z ∼ 9-10 universe based on HST+spitzer observations over 19 clash clusters: three candidate z ∼ 9-10 galaxies and improved constraints on the star formation rate density at Z ∼ 9.2. Astrophys. J. 795, 126 (2014).

[b7] Finkelstein S. L. . The evolution of the galaxy rest-frame ultraviolet luminosity function over the first two billion years. Preprint at arXiv: 1410.5439 (2014).

[b8] Planck Collaboration. . Planck 2015 results. XIII. Cosmological parameters. Preprint at arXiv: 1502.01589 (2015).

[b9] Santos M. R., Bromm V. & Kamionkowski M. The contribution of the first stars to the cosmic infrared background. Mon. Not. R. Astron. Soc. 336, 1082–1092 (2002).

[b10] Salvaterra R. & Ferrara A. The imprint of the cosmic dark ages on the near-infrared background. Mon. Not. R. Astron. Soc. 339, 973–982 (2003).

[b11] Cooray A., Bock J. J., Keatin B., Lange A. E. & Matsumoto T. First star signature in infrared background anisotropies. Astrophys. J. 606, 611–624 (2004).

[b12] Fernandez E. R., Komatsu E., Iliev I. T. & Shapiro P. R. The cosmic near-infrared background. II. fluctuations. Astrophys. J. 710, 1089–1110 (2010).

[b13] Kashlinsky A. . New measurements of the cosmic infrared background fluctuations in deep spitzer/IRAC survey data and their cosmological implications. Astrophys. J. 753, 63 (2012).

[b14] Matsumoto T. . AKARI Observation of the Fluctuation of the Near-infrared Background. Astrophys. J. 742, 124 (2011).

[b15] Cooray A. . Near-infrared background anisotropies from diffuse intrahalo light of galaxies. Nature 490, 514–516 (2012). 23099405 10.1038/nature11474

[b16] Zemcov M. . On the origin of near-infrared extragalactic background light anisotropy. Science 346, 732–735 (2014). 25378620 10.1126/science.1258168

[b17] Seo H. J. . AKARI Observation of the Sub-degree Scale Fluctuation of the Near-infrared Background. Astrophys. J. 807, 140 (2015).

[b18] Cappelluti N. . Cross-correlating Cosmic Infrared and X-Ray Background Fluctuations: Evidence of Significant Black Hole Populations among the CIB Sources. Astrophys. J. 769, 68 (2013).

[b19] Yue B., Ferrara A., Salvaterra R. & Chen X. The contribution of high-redshift galaxies to the near-infrared background. Mon. Not. R. Astron. Soc. 431, 383–393 (2013).

[b20] Helgason K., Ricotti M. & Kashlinsky A. Reconstructing the near-infrared background fluctuations from known galaxy populations using multiband measurements ofluminosity functions. Astrophys. J. 752, 113 (2012).

[b21] Grogin N. A. . CANDELS: the cosmic assembly near-infrared deep extragalactic legacy survey. Astrophys. J. Suppl. S. 197, 35 (2011).

[b22] Koekemoer A. M. . CANDELS: the cosmic assembly near-infrared deep extragalactic legacy survey-the hubble space telescope observations. Astrophys. J. Suppl. 197, 36 (2011).

[b23] Giavalisco M. . The great observatories origins deep survey: initial results from optical and near-infrared imaging. Astrophys. J. 600, L93–L98 (2004).

[b24] Windhorst R. A. . The hubble space telescope wide field camera 3 early release science data: panchromatic faint object counts for 0.2-2 fim Wavelength. Astrophys. J. Suppl. 193, 27 (2011).

[b25] Fixsen D. J., Moseley S. H. & Arendt R. G. Calibrating Array Detectors. Astrophys. J. Suppl. 128, 651–658 (2000).

[b26] Guo Y. . CANDELS multi-wavelength catalogs: source detection and photometry in the GOODS-south field. Astrophys. J. Suppl. 207, 24 (2013).

[b27] Amblard A. . Submillimetre galaxies reside in dark matter haloes with masses greater than3x10^11^ solar masses. Nature 470, 510–512 (2011). 21326201 10.1038/nature09771

[b28] Komatsu E. . Seven-year wilkinson microwave anisotropy probe (WMAP) observations: cosmological interpretation. Astrophys. J. Suppl. 192, 18 (2011).

[b29] Lin Y.-T., Mohr J. J. & Stanford S. A. K-Band Properties of Galaxy Clusters and Groups. Luminosity function, radial distribution, and halo occupation number. Astrophys. J. 610, 745–761 (2004).

[b30] Krick J. E. & Bernstein R. A. Diffuse Optical Light in Galaxy Clusters. II. Correlations with Cluster Properties. Astron. J. 134, 466–493 (2007).

[b31] Cooray A. & Sheth R. Halo models of large scale structure. Phys. Phys. Rep. 372, 1–129 (2002).

[b32] Navarro J. F., Frenk C. S. & White S. D. M. A universal density profile from hierarchical clustering. Astrophys. J. 490, 493–508 (1997).

[b33] Arendt R. G., Kashlinsky A., Moseley S. H. & Mather J. Cosmic Infrared Background Fluctuations in Deep Spitzer Infrared Array Camera Images: Data Processing and Analysis. Astrophys. J. Suppl. 186, 10–47 (2010).

[b34] Donnerstein R. L. The contribution of faint galaxy wings to source-subtracted near-infrared background fluctuations. Mon. Not. R. Astron. Soc. 449, 1291–1297 (2015).

[b35] Planck Collaboration. . Planck intermediate results. XXX. The angular power spectrum of polarized dust emission at intermediate and high Galactic latitudes. Preprint at arXiv: 1409.5738 (2014).

[b36] Cooray A., Gong Y., Smidt J. & Santos M. G. The near-infrared background intensity and anisotropies during the epoch of reionization. Astrophys. J. 756, 92 (2012).

[b37] Fernandez E. R., Iliev I. T., Komatsu E. & Shapiro P. R. The Cosmic near Infrared Background. III. Fluctuations. Astrophys. J. 750, 20 (2012).

[b38] Salpeter E. E. The luminosity function and stellar evolution. Astrophys. J. 121, 161 (1955).

[b39] Larson R. B. in The Stellar Initial Mass Function (ed. Nakamoto T. 336–340Star Formation (1999).

[b40] Tinker J. . Toward a halo mass function for precision cosmology: the limits of universality. Astrophys. J. 688, 709–728 (2008).

[b41] Zheng Z. . Theoretical models of the halo occupation distribution: separating central and satellite galaxies. Astrophys. J. 633, 791–809 (2005).

[b42] Metropolis N., Rosenbluth A. W., Rosenbluth M. N., Teller A. H. & Teller E. Equation of state calculations by fast computing machines. J. Chem. Phys. 21, 1087 (1953).

[b43] Hastings W. K. Monte Carlo sampling methods using Markov chains and their applications. Biometrika 57, 97 (1970).

[b44] Franceschini A., Rodighiero G. & Vaccari M. Extragalactic optical-infrared background radiation, its time evolution and the cosmic photon-photon opacity. Astron. Astraphys. 487, 837–852 (2008).

[b45] H.E.S.S. Collaboration. . Measurement of the extragalactic background light imprint on the spectra of the brightest blazars observed with H.E.S.S. Astron. Astraphys. 550, A4 (2013).

[b46] Atek H. . New constraints on the faint end of the UV luminosity function at z ∼ 7-8 using the gravitational lensing of the hubble frontier fields cluster A2744. Astrophys. J. 800, 18 (2015).

[b47] Robertson B. E. . New constraints on cosmic reionization from the 2012 hubble ultra deep field campaign. Astrophys. J. 768, 71 (2013).

[b48] Bouwens R. J. . UV luminosity functions at redshifts z ∼ 4 to z ∼ 10: 10,000 galaxies from HST legacy fields. Astrophys. J. 803, 34 (2015).

[b49] Kelsall T. . The COBE diffuse infrared background experiment search for the cosmic infrared background. ii. model of the interplanetary dust cloud. Astrophys. J. 508, 44–73 (1998).

[b50] Beckwith S. V. W. . The hubble ultra deep field. Astron. J. 132, 1729–1755 (2006).

[b51] Arendt R. G., Fixsen D. J. & Moseley S. H. in A practical demonstration of self-calibration of NICMOS HDF North and South Data vol. 281 (eds Bohlender, D. A., Durand, D. & Handley, T. H.) 217 (Astronomical Society of the Pacific Conference Series, 2002).

[b52] Kashlinsky A., Arendt R. G., Mather J. & Moseley S. H. Tracing the first stars with fluctuations of the cosmic infrared background. Nature 438, 45–50 (2005). 16267547 10.1038/nature04143

[b53] Hivon E. . MASTER of the cosmic microwave background anisotropy power spectrum: a fast method for statistical analysis of large and complex cosmic microwave background data sets. Astrophys. J. 567, 2–17 (2002).

[b54] Thompson R. I., Eisenstein D., Fan X., Rieke M. & Kennicutt R. C. Constraints on the cosmic near-infrared background excess from NICMOS deep field observations. Astrophys. J. 657, 669–680 (2007).

[b55] Witt A. N., Mandel S., Sell P. H., Dixon T. & Vijh U. P. Extended red emission in high galactic latitude interstellar clouds. Astrophys. J. 679, 497–511 (2008).

[b56] Paley E. S., Low F. J., McGraw J. T., Cutri R. M. & Rix H.-W. An infrared/optical investigation of 100 micron 'cirrus'. Astrophys. J. 376, 335–341 (1991).

[b57] Ienaka N. . Diffuse galactic light in the field of the translucent high galactic latitude cloud MBM32. Astrophys. J. 767, 80 (2013).

[b58] Guhathakurta P. & Tyson J. A. Optical characteristics of Galactic 100 micron cirrus. Astrophys. J. 346, 773–793 (1989).

[b59] Brandt T. D. & Draine B. T. The Spectrum of the diffuse galactic light: the milky way in scattered light. Astrophys. J. 744, 129 (2012).

[b60] Purcell C. W., Bullock J. S. & Zentner A. R. Shredded Galaxies as the Source of Diffuse Intrahalo Light on Varying Scales. Astrophys. J. 666, 20–33 (2007).

[b61] Gonzalez A. H., Zabludoff A. I. & Zaritsky D. Intracluster light in nearby galaxy clusters: relationship to the halos of brightest cluster galaxies. Astrophys. J. 618, 195–213 (2005).

[b62] Schiminovich D. . The GALEX-VVDS measurement of the evolution of the far-ultraviolet luminosity density and the cosmic star formation rate. Astrophys. J. 619, L47–L50 (2005).

[b63] Oesch P. A. . The evolution of the ultraviolet luminosity function from z ∼ 0.75 to z ∼ 2.5 using HST ERS WFC3/UVIS observations. Astrophys. J. 725, L150–L155 (2010).

[b64] Reddy N. A. & Steidel C. C. A steep faint-end slope of the uv luminosity function at z ∼ 2-3: implications for the global stellar mass density and star formation in low-mass halos. Astrophys. J. 692, 778–803 (2009).

[b65] McLure R. J. . A new multifield determination of the galaxy luminosity function at z**=**7-9 incorporating the 2012 Hubble ultra-deep field imaging. Mon. Not. R. Astron. Soc. 432, 2696–2716 (2013).

[b66] Kistler M. D., Yuksel H., Beacom J. F., Hopkins A. M. & Wyithe J. S. B. The star formation rate in the reionization era as indicated by gamma-ray bursts. Astrophys. J. 705, L104–L108 (2009).

[b67] Robertson B. E. & Ellis R. S. Connecting the gamma ray burst rate and the cosmic star formation history: implications for reionization and galaxy evolution. Astrophys. J. 744, 95 (2012).

[b68] Tanvir N. R. . Star formation in the early universe: beyond the tip of the iceberg. Astrophys. J. 754, 46 (2012).

[b69] Madau P., Haardt F. & Rees M. J. Radiative transfer in a clumpy universe. iii. the nature of cosmological ionizing sources. Astrophys. J. 514, 648–659 (1999).

