# Supplementary material for: Ultraviolet luminosity density of the universe during the epoch of reionization
Source: Nat Commun. 2015 Sep 8;6:7945. doi: 10.1038/ncomms8945 (PMC4569697; doi:10.1038/ncomms8945)
Supplement: Supplementary Information — Supplementary Figures 1-2, Supplementary Table 1 and Supplementary Note 1 [file ncomms8945-s1.pdf]

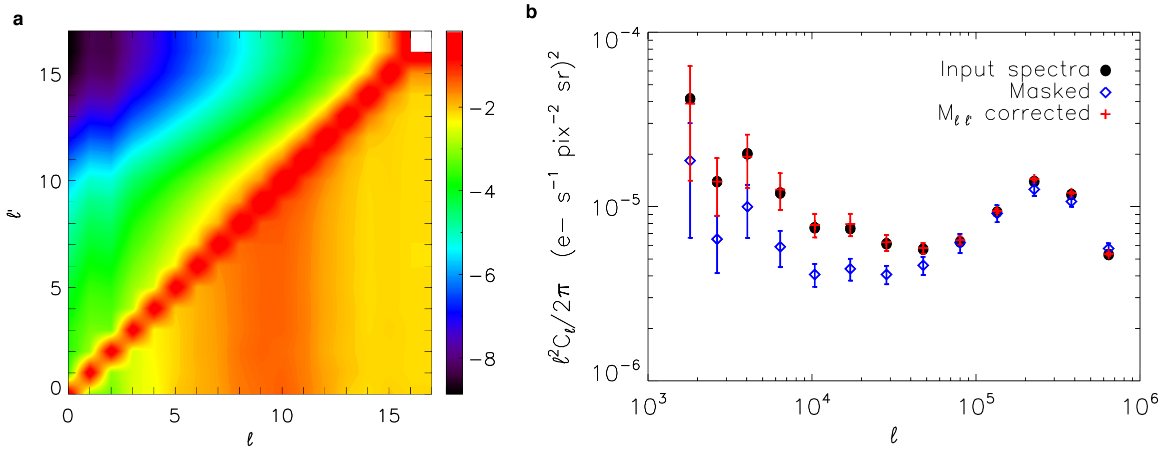

Supplementary Figure 1 | Mode-mode coupling correction. **a**, the mode-mode coupling matrix as generated from our source mask. **b**, $M_{\ell\ell'}$ validity simulation. We generate 90 Gaussian maps with a known input power spectrum (black filled circles). For each realization, we apply the mask to the simulated map and compute the resulting power spectrum (blue diamonds). Finally, we correct the masked power spectrum with our mode-mode coupling matrix to recover the input power spectrum (red crosses), which validates our masking correction.

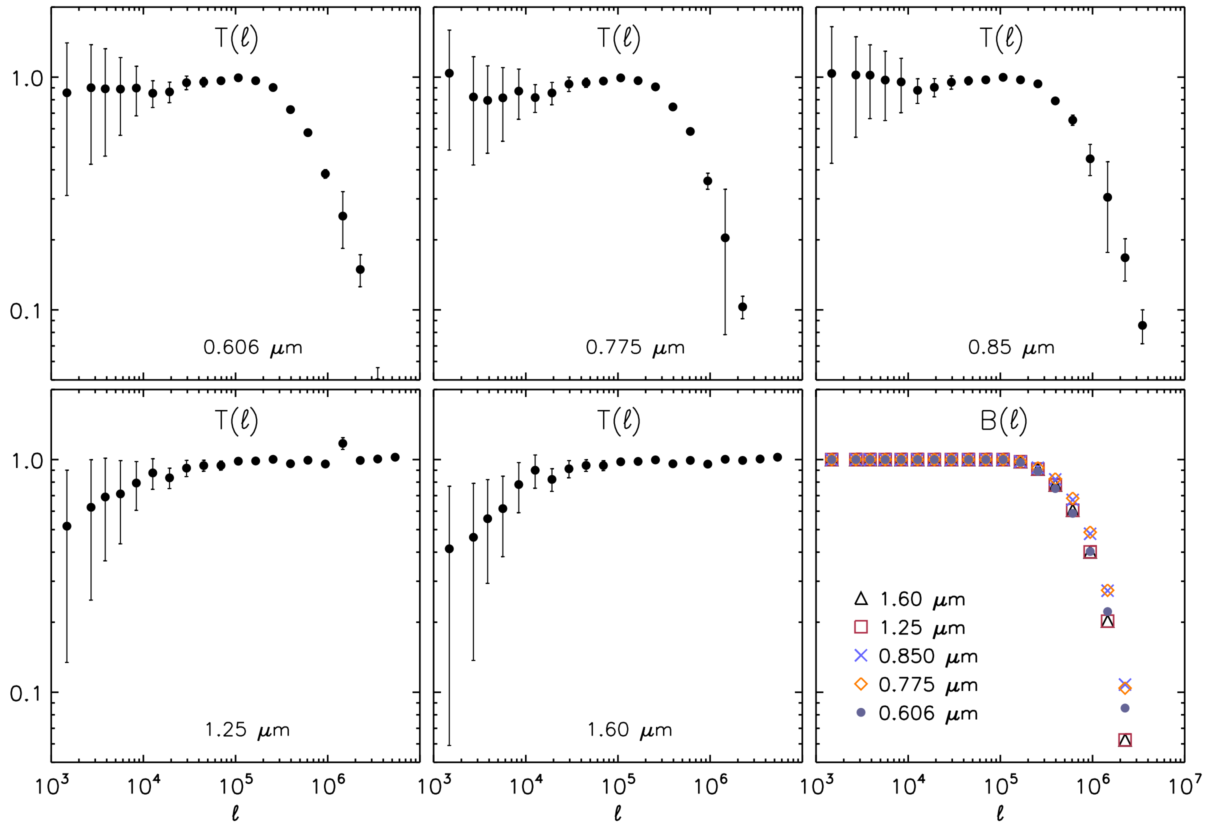

Supplementary Figure 2 | Transfer and beam functions. The T(ℓ)'s were generated from a minimum of 50 simulations in each band. These simulations incorporate the effects of the map-making algorithm, tiling pattern, varying exposure depths, residual (temporal) offsets, and cropping effects, specific to each filter. The beam transfer function, $B(\ell)$, in each band is just the PSF of each band in harmonic space.

ℓ	$\ell^2\,C_\ell^{1.6}\,/\,2\pi$	$\ell^2\,C_\ell^{1.25}\,/\,2\pi$	$\ell^2\,C_\ell^{0.850}\,/\,2\pi$	$\ell^2\,C_\ell^{0.775}\,/\,2\pi$	$\ell^2\,C_\ell^{0.606}\,/\,2\pi$
1.81×10^3	1.10 ± 1.17	1.10 ± 0.98	$(1.86 \pm 1.33) \times 10^{-1}$	$(2.30 \pm 1.69) \times 10^{-1}$	$(1.70 \pm 1.30) \times 10^{-1}$
2.64×10^3	$(8.30 \pm 6.60) \times 10^{-1}$	1.08 ± 0.72	$(9.55 \pm 5.10) \times 10^{-2}$	$(9.02 \pm 6.19) \times 10^{-2}$	$(9.01 \pm 5.38) \times 10^{-2}$
4.04×10^3	$(7.78 \pm 4.09) \times 10^{-1}$	$(7.94 \pm 4.04) \times 10^{-1}$	$(8.30 \pm 3.25) \times 10^{-2}$	$(7.21 \pm 3.29) \times 10^{-2}$	$(4.74 \pm 2.47) \times 10^{-2}$
6.40×10^3	$(6.33 \pm 2.30) \times 10^{-1}$	$(9.61 \pm 3.53) \times 10^{-1}$	$(9.61 \pm 3.23) \times 10^{-2}$	$(9.40 \pm 3.34) \times 10^{-2}$	$(3.29 \pm 1.17) \times 10^{-2}$
1.04×10^4	$(4.18 \pm 0.94) \times 10^{-1}$	$(6.60 \pm 1.43) \times 10^{-1}$	$(6.70 \pm 1.47) \times 10^{-2}$	$(3.98 \pm 0.91) \times 10^{-2}$	$(2.71 \pm 0.57) \times 10^{-2}$
1.71×10^4	$(4.01 \pm 0.63) \times 10^{-1}$	$(5.29 \pm 0.77) \times 10^{-1}$	$(3.13 \pm 0.41) \times 10^{-2}$	$(3.70 \pm 0.57) \times 10^{-2}$	$(1.86 \pm 0.27) \times 10^{-2}$
2.85×10^4	$(2.89 \pm 0.39) \times 10^{-1}$	$(3.71 \pm 0.46) \times 10^{-1}$	$(2.66 \pm 0.28) \times 10^{-2}$	$(3.02 \pm 0.39) \times 10^{-2}$	$(1.24 \pm 0.17) \times 10^{-2}$
4.76×10^4	$(2.76 \pm 0.37) \times 10^{-1}$	$(3.39 \pm 0.41) \times 10^{-1}$	$(2.90 \pm 0.27) \times 10^{-2}$	$(2.30 \pm 0.34) \times 10^{-2}$	$(9.62 \pm 1.74) \times 10^{-3}$
7.99×10^4	$(2.50 \pm 0.43) \times 10^{-1}$	$(2.85 \pm 0.46) \times 10^{-1}$	$(2.54 \pm 0.30) \times 10^{-2}$	$(1.92 \pm 0.40) \times 10^{-2}$	$(1.13 \pm 0.24) \times 10^{-2}$
1.34×10^5	$(3.19 \pm 0.56) \times 10^{-1}$	$(4.03 \pm 0.63) \times 10^{-1}$	$(3.76 \pm 0.45) \times 10^{-2}$	$(2.75 \pm 0.55) \times 10^{-2}$	$(1.67 \pm 0.34) \times 10^{-2}$
2.26×10^5	$(7.29 \pm 0.80) \times 10^{-1}$	$(8.50 \pm 0.89) \times 10^{-1}$	$(7.76 \pm 0.69) \times 10^{-2}$	$(5.16 \pm 0.74) \times 10^{-2}$	$(3.27 \pm 0.47) \times 10^{-2}$
3.82×10^5	1.62 ± 0.09	1.80 ± 0.11	$(2.33 \pm 0.13) \times 10^{-1}$	$(1.20 \pm 0.10) \times 10^{-1}$	$(8.96 \pm 0.75) \times 10^{-2}$
6.44×10^5	4.59 ± 0.13	4.96 ± 0.16	$(5.73 \pm 0.48) \times 10^{-1}$	$(3.24 \pm 0.22) \times 10^{-1}$	$(2.26 \pm 0.13) \times 10^{-1}$
1.09×10^6	$(1.49 \pm 0.02) \times 10^1$	$(1.55 \pm 0.05) \times 10^1$	2.31 ± 0.80	1.09 ± 0.47	$(7.08 \pm 0.70) \times 10^{-1}$

Supplementary Table 1 | Final HST power spectra. Corrected auto-spectra, $\ell^2 C_\ell/2\pi$ in units of (nW m^{-2} sr^{-1})^2, for 5 bands. The quoted errors are the 1 σ uncertainties.

Supplementary Note 1 | Data Availability: The self-calibrated mosaics used for the fluctuation study, including jack-knives with data separated to epochs, and the detected source mask, mode-coupling matrix, beam functions, and the transfer functions are available at `http://herschel.uci.edu/CANDELS`
